# RFamidergic neurons in the olfactory centers of the terrestrial slug *Limax*

**DOI:** 10.1186/s40851-018-0108-9

**Published:** 2018-08-09

**Authors:** Yuko Matsuo, Amami Yamanaka, Ryota Matsuo

**Affiliations:** Laboratory of Neurobiology, International College of Arts and Sciences, Fukuoka Women’s University, 1-1-1 Kasumigaoka, Higashi-ku, Fukuoka, 813-8529 Japan

**Keywords:** RFamide, Expression map, *Lehmannia*, Procerebral lobe, Field potential oscillation, FMRFamide, Pedal peptide

## Abstract

**Background:**

The terrestrial slug *Limax* has long been used as a model for the study of olfactory information processing and odor learning. Olfactory inputs from the olfactory epithelium are processed in the tentacular ganglion and then in the procerebrum. Glutamate and acetylcholine are the major neurotransmitters used in the procerebrum. Phe-Met-Arg-Phe-NH_2_ (FMRFamide) has been shown to be involved in the regulation of the network activity of the procerebrum. Although there are thought to be various RFamide family peptides other than FMRFamide that are potentially recognized by anti-FMRFamide antibody in the central nervous system of mollusks, identifying the entire repertoire of RFamide peptides in *Limax* has yet to be achieved.

**Methods:**

In the present study, we made a comprehensive search for RFamide peptide-encoding genes from the transcriptome data of *Limax*, and identified 12 genes. The expression maps of these RFamide genes were constructed by in situ hybridization in the cerebral ganglia including the procerebrum, and in the superior/inferior tentacles.

**Results:**

Ten of 12 genes were expressed in the procerebrum, and nine of 12 genes were expressed in the tentacular ganglia. Gly-Ser-Leu-Phe-Arg-Phe-NH_2_ (GSLFRFamide), which is encoded by two different genes, LFRFamide1 (Leu-Phe-Arg-Phe-NH_2_–1) and LFRFamide2 (Leu-Phe-Arg-Phe-NH_2_–2), decreased the oscillatory frequency of the local field potential oscillation in the procerebrum when exogenously applied in vitro. We also found by immunohistochemistry that the neurons expressing pedal peptide send efferent projections from the procerebrum back to the tentacular ganglion.

**Conclusion:**

Our findings suggest the involvement of a far wider variety of RFamide family peptides in the olfactory information processing in *Limax* than previously thought.

**Electronic supplementary material:**

The online version of this article (10.1186/s40851-018-0108-9) contains supplementary material, which is available to authorized users.

## Background

The terrestrial slug *Limax* has been used as a model in the study of olfactory information processing [16, 17, 19, 50]. It can form an associative olfactory memory in a single conditioning session. For example, the slug avoids the odor of a vegetable once it has been presented in combination with the bitter taste of quinidine sulfate solution [39, 50]. Most of the information of odor molecules received by olfactory receptors on the tip of the tentacle is conveyed to the tentacular ganglion (TG, a primary olfactory center) and then to the procerebrum (PC), a higher olfactory center in the brain containing more than 10^5^ interneurons [5, 6]. The learning ability is critically dependent on the PC [19, 27, 42, 63], and the change in the local field potential (LFP) oscillation recorded on the surface of the PC is thought to reflect the information processing of the odor that the slug previously learned [6, 24, 29, 48, 51].

The PC is thought to consist of two electrophysiologically distinct populations of interneurons, bursting and nonbursting neurons, and the oscillatory activity of the LFP has been explained by the mutual synaptic interaction between these two types of interneurons [31, 60]. The nonbursting neurons, the major component of the PC, receive olfactory inputs from the tentacular nerves as well as inhibitory input from the bursting neurons, which are a minor component of the PC. The LFP oscillation reflects periodic large inhibitory currents generated in the population of nonbursting neurons caused by the inputs from the bursting neurons nearby. Although there has been no conclusive demonstration, several lines of evidence suggest that glutamate and acetylcholine are used as neurotransmitters in the respective bursting and nonbursting neurons [18, 40, 44, 61, 62]. Therefore, the oscillatory activity of the LFP is believed to be realized by the crosstalk between cholinergic and glutamatergic neurons.

There are also other neurotransmitters that affect the oscillatory activity of the LFP in the PC, such as serotonin, dopamine, Phe-Met-Arg-Phe-NH_2_ (FMRFamide or FMRFa), histamine, octopamine, and noradrenaline, that change the oscillatory frequency when applied exogenously to the brain in vitro [18, 32, 45, 46]. Although many of these neurotransmitters send projections from outside of the PC, FMRFa is unique in that FMRFa-immunoreactive (FMRFa-IR) cell bodies have been identified both within and outside the PC [8, 32].

However, it has been pointed out that the specificity of anti-FMRFa antibodies is relatively low, and the antibodies are prone to react with the peptides whose C-terminus is -Arg-Phe-NH_2_, i.e. Arg-Phe-amides (RFamides, [20, 52, 59]). Indeed, most reports including histochemical staining in gastropods have cautiously used the expression “FMRFa-like IR” rather than “FMRFa IR” [8, 11, 36, 55, 58]. To address this issue, it will be necessary to determine the entire repertoire of RFamide peptides that are potentially recognized by anti-FMRFa antibody, and might affect the olfactory information processing in the PC.

Recently, a number of RFamide-family peptide genes have been identified or predicted in the central nervous system (CNS) of mollusks through transcriptome analyses [1, 2, 64]. These reports motivated us to analyze the expression of each RFamide gene species in and near the olfactory centers. In the present study, we identified 12 RFamide peptide-coding genes from the transcriptome data of the *Limax* CNS, including one gene that had not been considered an RFamide-encoding gene. We then determined their spatial expression patterns in the TGs and the cerebral ganglia (CG, including the PC) by in situ hybridization. We focused on these regions of the CNS because RFamidergic neurons located at these sites potentially contribute to the modulation of olfactory information processing. In addition, we analyzed the physiological activity of one of the peptides in the LFP oscillation in the PC, and also the projection patterns of the neurons expressing another peptide in the PC.

## Methods

### Animals

Terrestrial slugs *Limax valentianus* maintained in our laboratory as a closed colony (for at least 26 generations) were used in the present study. They were fed a diet of humidified powder mixture (see [14] for composition). As the slugs were kept in an incubator (19 °C) equipped with a window in our laboratory, they were almost under natural light-dark cycles.

### Transcriptome analysis

Transcriptome data was obtained as follows (NCBI BioProject acc. no. PRJDB3972): The total RNAs were extracted from the heart and the visceral giant cell [43] of the slugs by the acid guanidinium thiocyanate-phenol-chloroform method [7]. The cDNA libraries were constructed using a SureSelect strand specific RNA library prep kit (Agilent, Santa Clara, CA, USA) following the manufacturer’s instructions, and sequencing was performed with 100 bp pair-end reads (> 30 million reads) using the Illumina Hiseq 2500 platform. The sequences were de novo assembled using a program Trinity [15].

### Identification of RFamide-encoding transcripts

Most of the genes that we report in the present study were identified in the transcriptome data of *Limax* (see above) as homologous cDNAs of those reported in *Deroceras reticulatum* [1]. However, we also searched for unidentified cDNA data encoding RFamide peptides. All cDNA data were computationally translated in three frames using a web-based program (http://www.gen-info.osaka-u.ac.jp/~uhmin/study/orf/index.cgi), and we searched for the typical signatures of RFamide, i.e. Arg-Phe-Gly-Arg/Lys-Arg/Lys in the predicted amino acid sequences of propeptides. Of the positive hits, only the polypeptides with meaningful length (> 20 amino acids) were selected, and then tested for homology with the RFamide genes already reported by Ahn et al. [1]. Signal sequences were predicted using the web-based program SignalP 4.1 (http://www.cbs.dtu.dk/services/SignalP/).

### Molecular cloning and expression analysis of RFamide cDNAs

Partial cDNAs of the RFamide genes were cloned by reverse transcription-PCR (RT-PCR) using RNAs derived from the CNS of *Limax*, as described previously [14], except that RNAs were reverse-transcribed using oligo-dT primers. The nucleotide sequences of the PCR primers were designed based on the transcriptome data of *Limax* (see above), and are shown in Table 1. The amplicons were purified using the Wizard SV Gel and PCR clean-up system (Promega, Madison, WI, USA), and phosphorylated by T4 polynucleotide kinase (Takara, Ohtsu, Japan). The amplicons were ligated into a cloning vector pBluescriptII (KS) that had been digested with EcoRV followed by dephosphorylation by shrimp alkaline phosphatase (Takara). Competent *E. coli.* (Dh5α) was transformed with the ligated product, and the plasmids were obtained from the transformed *E. coli*. The direction and the nucleotide sequence of the insert cDNAs were determined by DNA sequencing. For the expression analysis of the mRNAs, we used the same set of PCR primers as those used for molecular cloning (Table 1). The numbers of the PCR cycles were 23–29 except for 18S rRNA (13 cycles). The PCR products were electrophoresed in 1% agarose gel, and were visualized using 0.5 μg/ml ethidium bromide under a UV illuminator.

**Table 1:** PCR primers used for the expression analysis and the preparation of the templates for the cRNA probes used in in situ hybridization

Target cDNA	Upstream primer	Downstream primer	Region	Genbank acc. no.	aa. identitiy (%) to *Deroceras* gene
FMRFamide	agacgataagctccaccgctttg	tcttcagcctgaacgtatgacgc	83–300 (2–219)	AB546959 (AB546960)	79.1
LFRFamide1	atgatcaagagtcagctctcag	atcgctgtcactaccccagtg	15–563	LC375313	83.1
LFRFamide2	cgactctgataaaaggggctcc	atgtgactactgggaacgccag	566–1021	LC375314	70.6
CCK/SK1	cgctgcccacactgaacaag	ggtttcattgttgttggcgaag	743–1111	LC375319	73.5
CCK/SK2	agagcacaccactgaaggac	tcggatgtgtcggggttta	451–912	LC375320	82.4
NP-F1	gttgctctaataatttgctcgggag	gccgccatgtttgttagttgac	303–782	LC375315	91.7
NP-F2	caacacactacgcccacacag	ctgttcacgagtgtgctcgag	972–1520	LC375316	92.9
pedal peptide	atagaatcgctggtgcgtcgag	cgccgttatcgctgagttcatg	4219–4716	LC375323	–
FaRP-A	tgacgagctcgatacgacctatg	cctcgtggtcaaaccttctgaac	399–967	LC375321	–
FaRP-B	agaccagcgtgcgcaccag	ctgggccagcagacattgac	144–650	LC375322	–
luqin1	ccacccttaagtcagcaactc	ggaatagaaacacaggagagcgatg	401–1051	LC375317	88.7
luqin2	ctcttgtctcatctgcccacac	agctatccgaaccgtgtgagtc	19–552	LC375318	65.4
18S rRNA	gcttaccaagctccgaccctcgtgg	cgtcactacctccccgtgccggg	62–383	LC375324	–

The accession numbers of the *Limax* RFamide cDNAs and the amino acid identities of precursors to the corresponding *Deroceras* homologues are shown in the right columns. The numerals in the “region” column indicate the amplified cDNA regions in the sequence data deposited in Genbank. The information for the splice variant of FMRFamide (FMRFamide Type II) is shown in parentheses

### Molecular cloning of pedal peptide cDNA

**Fig. 1:**
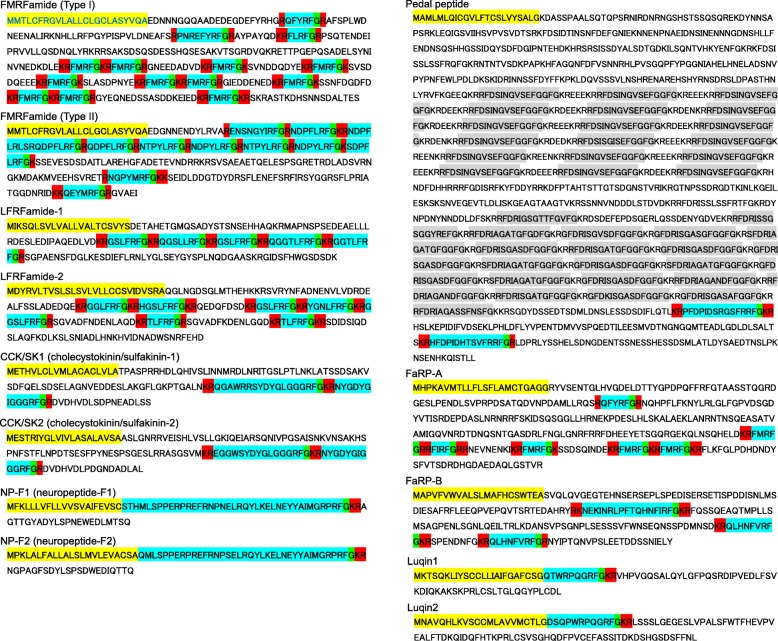
Predicted amino acid sequences of the precursor proteins encoding RFamide peptides. Only RFamide peptides are indicated, and other non-RFamide peptides are not highlighted (except for PP). Predicted RFamides are highlighted in blue, and predicted signal sequences are highlighted in yellow. Highlighted in red are the basic amino acids predicted to be the cleavage sites. Highlighted in green are the glycine residues used for C-terminal amidation. Highly repeated peptide sequences predicted to be encoded in PP mRNA are highlighted in gray

During the search for the data of cDNAs encoding unidentified RFamides as described above, we found a partial sequence of pedal peptide (PP)-like cDNA that was predicted to encode RFamide peptides in the transcriptome data of *Limax*. But the full-length cDNA of PP was not found in any databases including the transcriptome data of *Limax*, or in the report on the neuropeptide catalogue of *Deroceras* [1]. On the other hand, we have accidentally obtained a partial cDNA clone (Genbank acc. no. LC375774) that exhibits a very similar expression pattern in the brain and the superior tentacle (ST) during our in situ hybridization assay of peptide-encoding transcripts (unpublished observation). Moreover, the predicted amino acid sequence encoded in this transcript terminated as “RFDSINGVSE”, which is similar to the amino acid sequence that appeared repeatedly in the downstream PP clone (see Fig. 1). Since the neuropeptides of gastropods are often encoded tandemly in multiple copies in a single open reading frame [37, 54, 56], we speculated that this clone corresponds to the upstream region of PP cDNA. We thus designed two pairs of nested PCR primers to obtain the longer cDNA clone of PP by RT-PCR: the downstream primers were directed to the downstream partial PP, while the upstream primers were directed to the putative upstream clone described above. The nucleotide sequences of the PCR primers follow: the upstream primers were 5′-CACACAGGGAAAACGCGAGAGAACACTC-3′ (external) and 5′-GGCGAGGAGCAAAAGAGGAGATTCG-3′ (internal), and the downstream primers were 5′-GCCGCTTTCCAAACCCGCCAAACGCGGATG-3′ (external) and 5′-ACCACCGAAGCCAGTTGCGCCAGC-3′ (internal). The amplicon was ligated into the cloning vector pBluescriptII (KS) as described above and the plasmid was obtained. The nucleotide sequence of the insert cDNA was determined by DNA sequencing.

### In situ hybridization

The plasmids obtained for 12 genes were linearized by digestion with restriction enzymes appropriate for each cDNA clone, and cRNA probes were generated by in vitro transcription in the presence of digoxigenin-UTP as described previously [14]. The cRNA probes were titrated, and their working concentrations were determined so that antisense and sense probes would have equivalent titers. The brain and the superior/inferior tentacles (ST/IT) were dissected out from a slug deeply anesthetized by an injection of ice-cold Mg^2+^ buffer (57.6 mM MgCl_2_, 5.0 mM glucose, 5.0 mM HEPES, pH 7.0) into the body cavity, and were frozen in FSC22 Clear frozen section compound (Leica Biosystems, Nussloch, Germany) using liquid nitrogen. The sections (14 μm-thick) were cut in a cryostat, and mounted onto a glass slide coated with Vectabond (Vector Laboratories, Burlingame, CA). The hybridization and wash were performed as described previously using anti-digoxigenin antibody conjugated with alkaline phosphatase (Roche Diagnostics, Basel, Switzerland, [14]). For the colorimetric in situ hybridization, the signal was detected using 5-bromo-4-chloro-3-indoyl phosphate and nitro blue tetrazolium as described in Fukunaga et al. [14]. For florescence in situ hybridization, the signal was detected using a 2-hydroxy-3-naphtonic acid-2′-phenylanilide phosphate (HNPP) fluorescent detection set (Roche Diagnostics) according to the manufacturer’s instructions. We used the fluorescence in situ hybridization for the ST/IT samples because these tissues are innately brown-colored, which makes it difficult to distinguish positive hybridization signals using a colorimetric method. The images were acquired with an Eclipse E600 microscope (Nikon, Tokyo, Japan) equipped with a DP70 CCD camera (Olympus, Tokyo, Japan) and a × 10 (NA 0.30) or a × 20 (NA 0.50) objective lens.

### Antibodies and pre-adsorption experiment

Anti-pan-RFamide mouse monoclonal antibody was the generous gift of Dr. Shun Hamada [35], and was used at 1:200 dilution in the immunohistochemistry. The pan-RFamide antibody was generated by immunizing a mouse with Arg-Phe-NH_2_ conjugated to keyhole limpet hemocyanin via Cys residue, and it recognizes the peptides that terminate as RFamide in their C-termini. The specificity of this antibody had been confirmed by competitive ELISA [35]. Anti-PP antiserum was raised by immunization of a rabbit with peptide (CGFDRISGASDFGGFG) conjugated to keyhole limpet hemocyanin via N-terminal Cys residue. This peptide was non-RFamide peptide encoded repeatedly in the C-terminal half of the PP precursor, and was chosen so that we could prevent the antiserum from reacting to the C-terminal epitope (RFamide). The antiserum was used at 1:5000 in our immunohistochemistry. The specificity of the anti-PP antiserum was checked by pre-adsorption by incubating the diluted antiserum with 100 μM peptide (GFDRISGASDFGGFamide) at 4 °C overnight prior to primary antibody application.

### Immunohistochemistry

The brain or the ST/IT was dissected out under deep anesthesia as described above. They were fixed in 4% paraformaldehyde dissolved in PBS for 1 h at room temperature, and cryoprotected by incubation in 20% sucrose dissolved in PBS at 4 °C overnight. The tissues were frozen in FSC22 Clear frozen section compound using liquid nitrogen. The sections (14 μm-thick) were cut in a cryostat, and mounted onto a glass slide coated with Vectabond. After treatment with 5% neutralized formalin (10% neutralized formalin (Nakarai-Tesque, Kyoto, Japan) diluted into a half concentration with PBS) for 20 min, the sections were treated with 0.1% Triton X-100 in PBS (PBST) for 10 min, followed by a brief wash in PBS. After blocking in blocking buffer (2.5% BSA, 2.5% goat serum in PBST) for 1–7 h at room temperature, the sections were incubated with antibodies (see above) diluted with blocking buffer at 4 °C overnight. After washingthree times in PBS, the sections were incubated with the appropriate secondary antibodies (1:500 in blocking buffer) labeled with fluorophore (anti-mouse IgG antibody labeled with Alexa594 and/or anti-rabbit IgG antibody labeled with Alexa488, Thermo Fisher Scientific, Waltham, MA) for 1 h at room temperature. After a wash in PBS, the sections were incubated with 0.1 μg/ml 4′6-diamidino-2-phenylindole (DAPI) in PBS for 15 min, followed by a wash in PBS. The sections were coverslipped with Fluoromount-G (SouthernBiotech, Birmingham, AL, USA). The images were acquired with an Eclipse E600 microscope equipped with a DP70 CCD camera and a × 20 (NA 0.50) objective lens.

### Double staining with neurobiotin and anti-RFamide or anti-PP antibody

Neurobiotin (NB) was incorporated from the cut end of the superior tentacular nerve of an isolated CNS as described previously with a slight modification [44]. Briefly, NB was dissolved in physiological saline (70 mM NaCl, 2.0 mM KCl, 4.7 mM MgCl_2_, 4.9 mM CaCl_2_, 5.0 mM glucose, 5.0 mM HEPES, pH 7.0) to a concentration of 1%, and the solution was diluted to half the concentration (0.5%) with H_2_O. The cut end of the left superior tentacular nerve was suctioned with a glass capillary tube filled with the NB solution. The preparation was kept at 14 °C overnight, and then the brain was fixed in 4% paraformaldehyde in PBS for 1 h at room temperature. After cryoprotection with 20% sucrose (in PBS) for 4 °C overnight, the brain was frozen in FSC22 Clear frozen section compound using liquid nitrogen. The sectioning and immunohistochemical staining were performed as described above, except that Alexa488-labeled streptavidin (1:1000, Thermo Fisher Scientific) was added to the secondary antibody (1:500, Alexa568-labeled anti-rabbit antibody or Alexa594-labeled anti-mouse antibody).

### LFP recordings

The LFP oscillation on the surface of the PC was recorded extracellularly as described previously [45]. The oscillatory frequency was calculated based on the number of peaks during 2 min time frames. The samples whose LFP oscillatory frequency exceeded 0.82 Hz or was below 0.5 Hz at the start of the recording were not used for further recording. All the recordings were performed at 20–25 °C.

### Statistical analysis

The LFP oscillation data were statistically analyzed at each time point using a Student’s *t*-test with a significance level of *P* < 0.05. The data are expressed as the mean ± S.E. throughout.

## Results

### Identification of RFamide-encoding mRNAs

In the *Limax* transcriptome data, we searched for homologues of the genes that were predicted to encode RFamides in the other slug species *Deroceras reticulatum* [1]. Essentially, we followed the nomenclature of Ahn et al. [1]. However, FaRP-B (FMRFamide-related peptide-B) was newly named in this study, as this gene was only moderately similar to FaRP2 of *Deroceras* (Genbank acc. no. ARS01394, 53% identical to FaRP-B of *Limax* at the amino acid sequence level), making it difficult to judge whether it is an orthologue. See also Table 1 showing the identity (%) of the amino acids of the precursor protein between the counterparts from the two species.

We also independently searched for the data of transcripts that are predicted to encode a protein containing the sequence “RFG(R/K)(R/K)” in either of the three open reading frames, and identified two genes that were not identified or recognized as RFamide-encoding genes by Ahn et al. [1] (FaRP-A and PP, Fig. 1). FaRP-A (FMRFamide-related peptide-A) was predicted to encode four copies of canonical FMRFa peptides, but the primary structure of the precursor protein did not resemble any of the RFamide precursors of *Deroceras*, i.e., the most similar data of *Deroceras* was its FMRFamide (ARS01392) with only 26.5% amino acid identity [1]. On the other hand, the obvious orthologue of FMRFamide in *Limax valentianus* has already been identified (Fig. 1 and [32]). Therefore, we newly named this protein FaRP-A.

The precursor of PP was tentatively named as such because the amino acid sequence of the C-terminal half was similar to the pedal peptide of *Lymnaea* (Genbank acc no. AAP57098, [21]), although it is not certain whether they are actually orthologous to each other. The PP precursor was an unexpectedly large protein, and was predicted to encode 42 peptides. Only two of 42 peptides were predicted to contain “RFamide” in their C-termini (Fig. 1).

Because FaRP1 of *Deroceras* (Genbank acc. no. KY659292, [1]) seemed to be a splice variant of FMRFa, sharing the first exon with the canonical FMRFa (Type II FMRFa in [32]), we hereafter analyzed the expression of this transcript together with FMRFa (Type I FMRFa) by focusing on the expression of the first shared exon. Therefore, we further analyzed the expression patterns for 12 of the 13 genes listed in Fig. 1.

### Expression of RFamide-encoding mRNAs

**Fig. 2:**
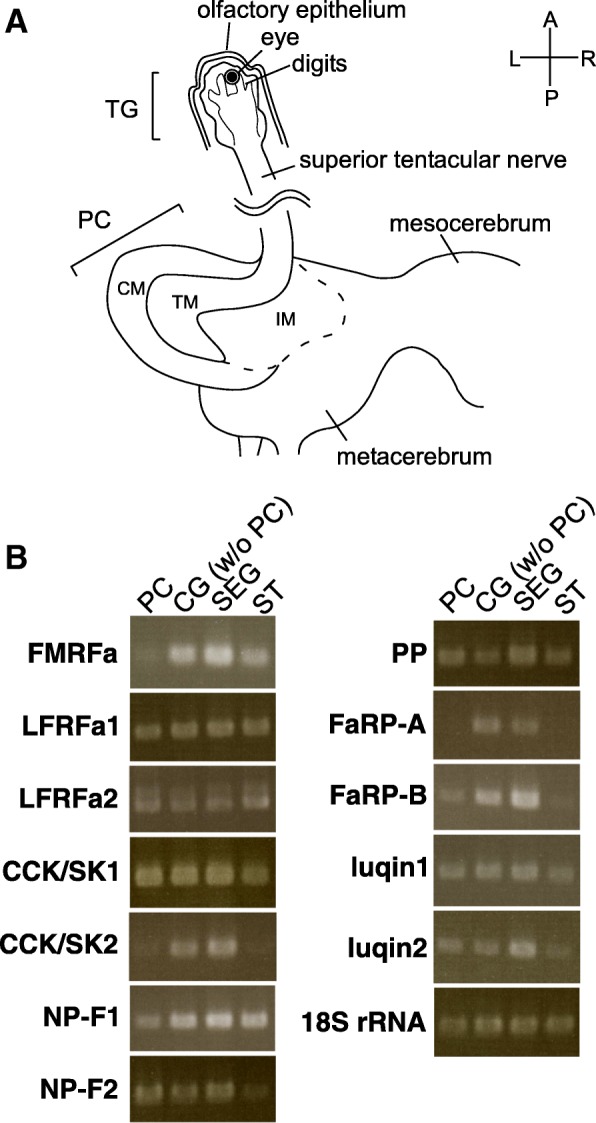
**a** A schema of the CG and the ST explaining the structures and the names of the CNS regions pertinent to the present study. Because the IT projects to the TM of the PC from the ventral side, it is not depicted in this figure. TG, tentacular ganglion; PC, procerebrum; CM, cell mass; TM, terminal mass; IM, internal mass. R, right; L, left; A, anterior; P, posterior. **b** RT-PCR for the 12 RFamide genes using the cDNA templates derived from 4 different parts of the CNS. For the FMRFa gene, the amplicon corresponds to the first exon common to the two splice variants. 18S rRNA serves as an internal control for the template cDNA amount. PC, procerebrum; CG (w/o PC), cerebral ganglia without the PC; SEG, subesophageal ganglia; ST, superior tentacle

To roughly analyze the expression profiles of the RFamide genes, each gene was amplified by RT-PCR using the mRNAs derived from 4 different parts of the CNS (Fig. 2a). All of the 12 genes were detected in the CG and subesophageal ganglia (SEG) (Fig. 2b). However, for some of them, the relative expression level was lower in the PC (FMRFa, CCK/SK2, FaRP-A, FaRP-B) and in the ST (CCK/SK2, NP-F2, FaRP-A, FaRP-B, luqin2). To further examine the expression of RFamide genes, we performed in situ hybridization in the brain and ST/IT.

### Expression mapping of RFamide-encoding mRNAs

**Fig. 3:**
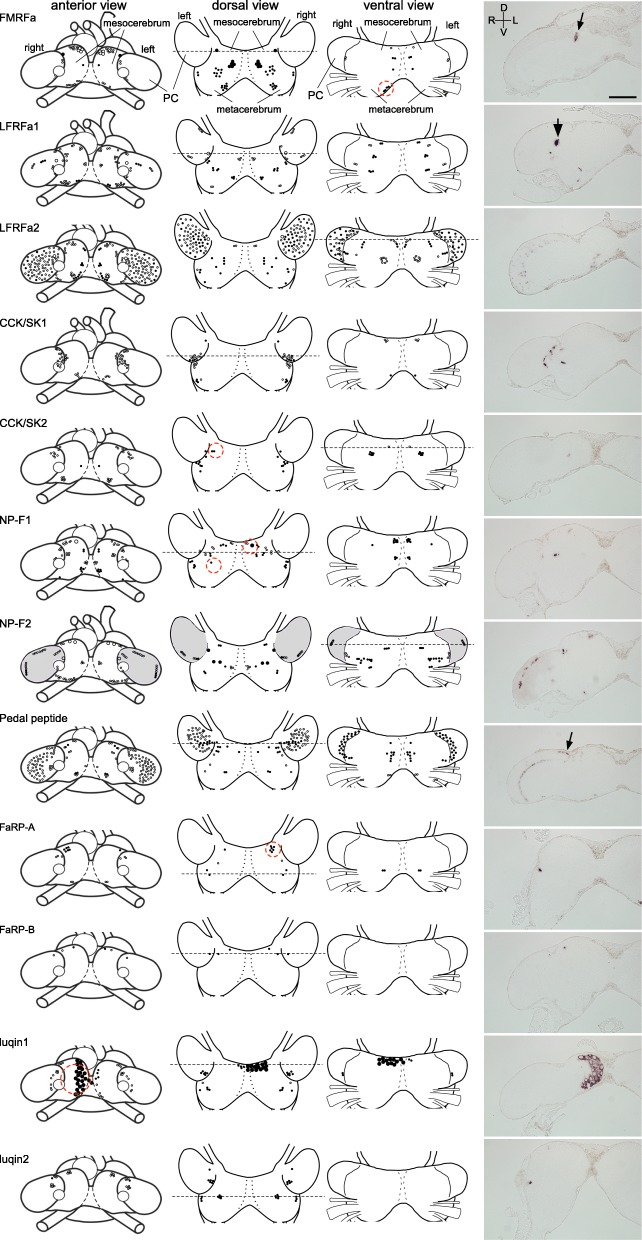
The expression maps of 12 RFamide genes in the cerebral ganglia. Open and closed circles indicate the cell bodies located in the deep and near the surface of the cerebral ganglia, respectively. Large neurons are indicated by large circles. The leftmost schemata indicate the anterior views of the brain and all the positively stained neurons are plotted. The next right two schemata show the dorsal and ventral views, and each neuron is assigned to one of these two schemata depending on the position relative to a dorso-ventral axis. The number of circles does not necessarily reflect the exact number of positive neurons, except as noted in Table 2. Numerous PC neurons expressing NP-F2 mRNA are shown in gray shade. Asymmetrically distributed neurons are circled with a red broken line for FMRFa, CCK/SK2, NP-F1, FaRP-A, and luqin1 in the schema. The photographs on the right are the representative images of *in situ* hybridization (coronal sections, i.e. anterior view) with antisense probes. The sectioning planes corresponding to the photographs on the right are indicated by dotted lines in the schema of the brain. Scale bar: 100 μm (applicable to all photographs). D, dorsal; V, ventral; R, right; L, left. Comments on the localization of each gene are shown in Table 2

**Fig. 4:**
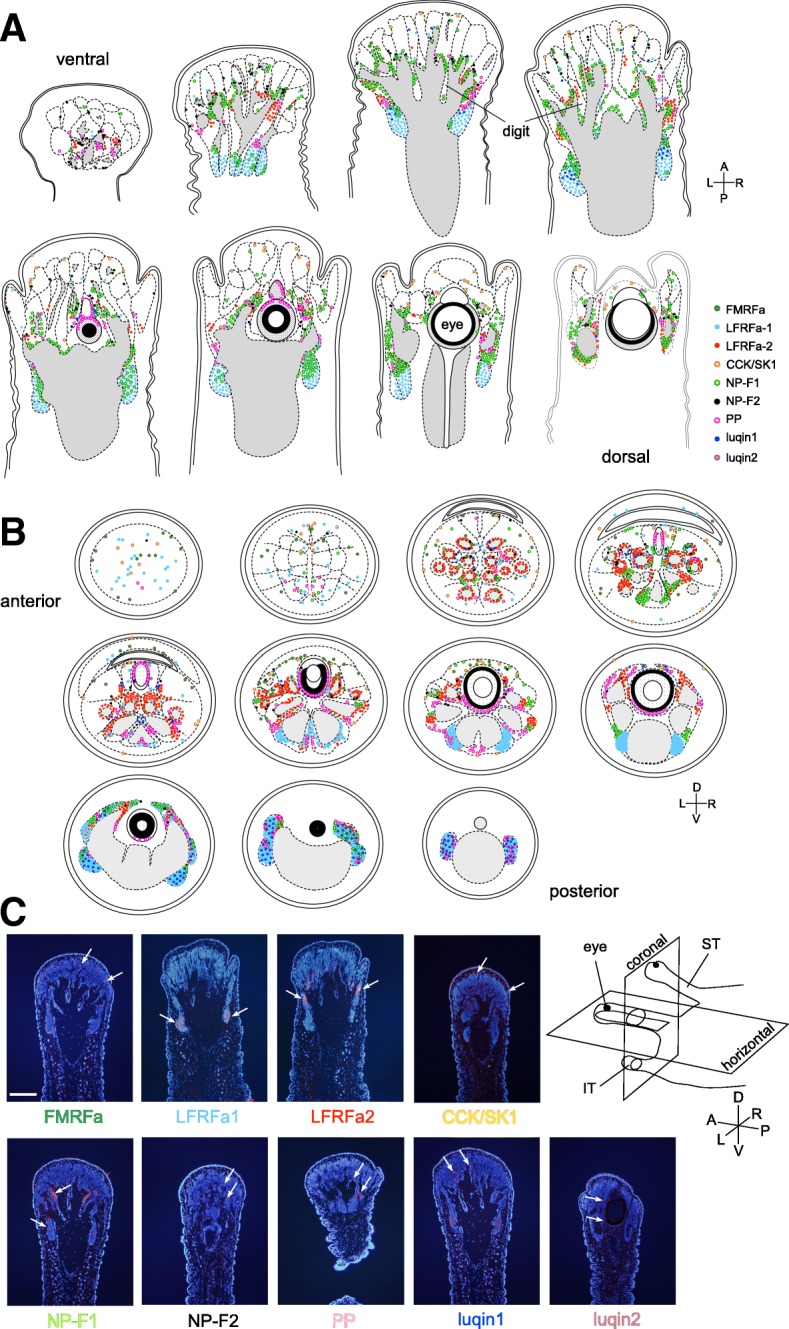
The expression maps of 9 RFamide genes in the ST. Schemata showing the distribution of the neurons expressing RFamide genes in (**a**) horizontal and (**b**) coronal sections. **c** Examples of images of in situ hybridization in horizontal sections of the left ST. Positive signals are in red, and nuclei are in blue. White arrows indicate some of the positive signals. The cartoon on the right explains the direction of the sectioning plane. Scale bars: 200 μm (applicable to all photographs). D, dorsal; V, ventral; A, anterior; P, posterior; R, right; L, left

**Fig. 5:**
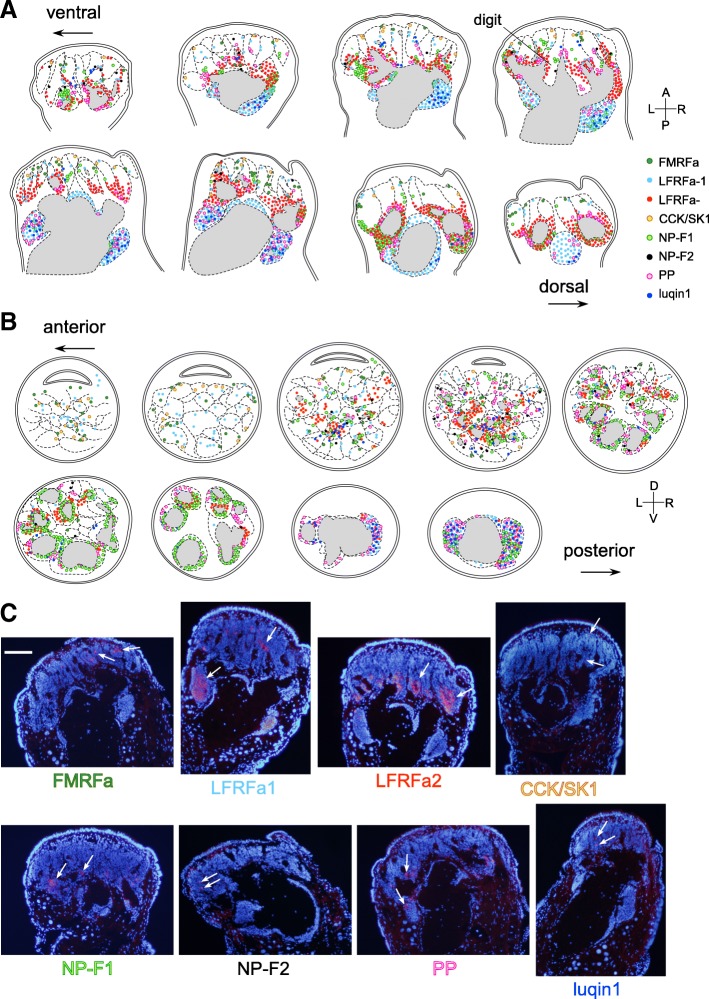
Expression maps of eight RFamide genes in the IT. Schemata showing the distribution of the neurons expressing RFamide genes in (**a**) horizontal and (**b**) coronal sections. **c** Examples of images of in situ hybridization in horizontal sections of the left IT. Positive signals are in red, and nuclei are in blue. White arrows indicate some of the positive signals. For the direction of the sectioning, see the cartoon in Fig. 4. Scale bars: 100 μm (applicable to all photographs). D, dorsal; V, ventral; A, anterior; P, posterior; R, right; L, left

The distribution of the cell bodies expressing each mRNA species was mapped in the CG (including the PC) and the ST/IT by in situ hybridization in the serial sections. For the CG, the distributions of the cell bodies of each mRNA were separately mapped on the schemata viewed from three different angles (anterior, dorsal, and ventral views, Fig. 3). For the ST and IT, the distributions were mapped together in the same schemata of the sections (approx. 70 μm apart from each other, Figs 4 and 5). The specificity of the cRNA probes was confirmed from the absence of signals when hybridized with sense probes in the adjoining sections (Additional file 1: Figure S1). The characteristics of the expression patterns of each gene are summarized in Table 2, focusing on (1) the abundance/distribution in the CG, (2) the right-left symmetry in the CG, (3) the characteristic expression pattern in the CG, and (4) the abundance/distribution in the ST/IT.

**Table 2:** Summary of the localization of 12 RFamide genes in the CG and ST/IT

	Abundance and distribution in the CG (Fig. 3)	Right-left symmetry (Fig. 3)	Characteristic expression pattern in the CG (Fig. 3)	Abundance and distribution in the ST/IT (Figs. 4 & 5)
*FMRFamide*	Many in the dorsal aspect of the CG. No positives in the PC	Asymmetrical (A cluster of neurons in the ventro-posterior region of the right CG)	A pair of large neurons (probably C3 motor neurons, [9]) in the dorso-anterior aspect of the PC	Scattered in the marginal area, away from the digit
*LFRFamide1*	Many in the CG (including the PC)	Symmetrical	A pair of large neurons at the basal region of the PC	Expressed in a majority of neurons in the cell mass at the basal region
*LFRFamide2*	Many in the CG (especially abundant the PC cell mass layer)	Symmetrical	A pair of large neurons at the basal region of the PC	Many along the borderline between the digits and neuropiles
*CCK/SK1*	Many in the basal region of the PC	Symmetrical		Found in the apical region
*CCK/SK2*	Most of the positives were localized to the CG other than the PC	Asymmetrical (Two neurons in the dorsal aspect of the left CG)	A pair of positives at the basal region of the PC	No positives in the ST/IT
*NP-F1*	Most of the positives were localized to the CG other than the PC	Asymmetrical (One large and one small positives located in the dorsal aspect of the respective right and left CG)	A pair of positives at the basal region of the PC	Localized primarily in the cell mass in and near the digits
*NP-F2*	Expression in seemingly all the PC neurons, and in several neurons in the other CG regions	Symmetrical	Conspicuous signals aligned in two rows in the PC	Localized to near the apical region
*pedal peptide*	Many positives in the PC, and a smaller number in the other CG regions	Symmetrical	Many positives along the edge of the cell mass and terminal mass layers of the PC	Localized to the digits cell mass, with some positives found in the putative photoreceptors of an eye
*FaRP-A*	A small number in the CG, but none in the PC	Asymmetrical (a culster located in the dorsal aspect of the right CG)	A small number of positives	No positives in the ST/IT
*FaRP-B*	Only 3 pairs of positives in the CG, including one pair in the basal region of the PC	Symmetrical	All 6 positives reside in the dorsal aspect	No positives in the ST/IT
*luqin1*	Positive neurons in the mesocerebrum, dorsal aspect of the metacerebrum, and in the basal region of the PC	Asymmetrical (larger number in the right mesocerebrum)	Many large positives in the mesocerebrum.	Scattered in the cell mass at the basal region, and also in the apical region
*luqin2*	A cluster of 5–9 positives in the dorsal aspect of the matacerebrum, and a cluster of 5–7 positives in the basal region of the PC	Symmetrical	No positives in the ventral aspect	A few positives in the anterior to an eye in the ST, whereas no positives in the IT

### Effect of GSLFRFamide on the LFP oscillation

**Fig. 6:**
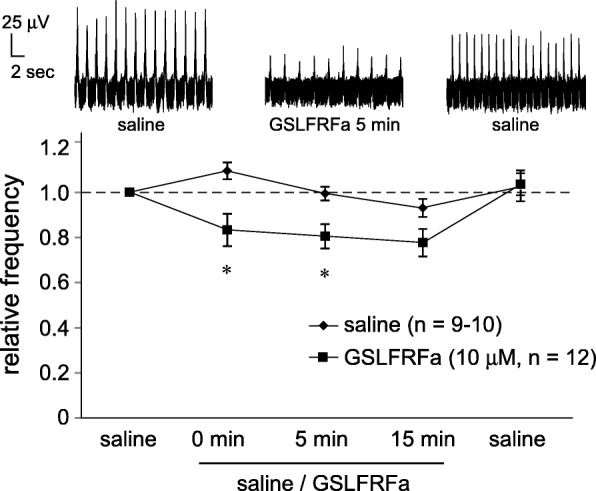
GSLFRFamide reduces the oscillatory frequency of LFP in the PC. The upper traces indicate the representative waveforms of LFP for 20 s before the application, during the presence, and after the removal of 10 μM GSLFRFa. *P < 0.05 vs. saline control

We next focused on GSLFRFamide, because it is predicted to be commonly encoded by two different transcripts, LFRFa1 and LFRFa2 (Fig. 1), both of which show substantially high expression levels in and near the PC (Figs. 2b and 3). In vitro application of 10 μM of GSLFRFamide reduced the oscillatory frequency of LFP recorded on the surface of the PC (*P* = 0.0065, 0.0072, and 0.057 for respectively 0 min, 5 min, and 15 min following GSLFRFamide application), and the frequency immediately returned to the baseline level just following the removal of the peptide (Fig. 6).

### RFamide peptides in the CG and the TGs

To see whether the RFamide peptides encoded by above mentioned transcripts are actually identifiable by immunohistochemical staining with anti-pan RFamide antibody, we investigated the distribution of RFamide peptides’ IR. The neuropil regions were densely stained, but many cell bodies in the CG (metacerebrum and mesocerebrum) and the PC were also positively stained (arrowheads in Additional file 2: Figure S2). IR was observed in the superior tentacular nerves (double white arrows in Additional file 2: Figure S2C, E, G, I, K), which is similar to the result in our previous immunohistochemical study using anti-FMRFa antibody [32, 41]. But we did not find strong IR signals in the neuropil regions (terminal and internal mass layers) of the PC (Additional file 2: Figure S2A-L). Judging from their localization, we also found the cell bodies of the neurons that were supposed to be luqin1-, NP-F2- and PP-expressing neurons (“X”, “Y”, and “Z” in Additional file 2: Figure S2I-N, see also Fig. 3).

We also immunostained RFamide peptides in the ST and IT that contain TGs, the primary olfactory center. Positively stained cell bodies were scattered in the cell body regions as well as just beneath the olfactory epithelium (white arrows and arrowheads in Additional file 3: Figure S3). Superior and inferior tentacular nerves also exhibited IR signals (double white arrows in Additional file 3: Figure S3).

### Projection patterns of RFamidergic neurons in the olfactory center

**Fig. 7:**
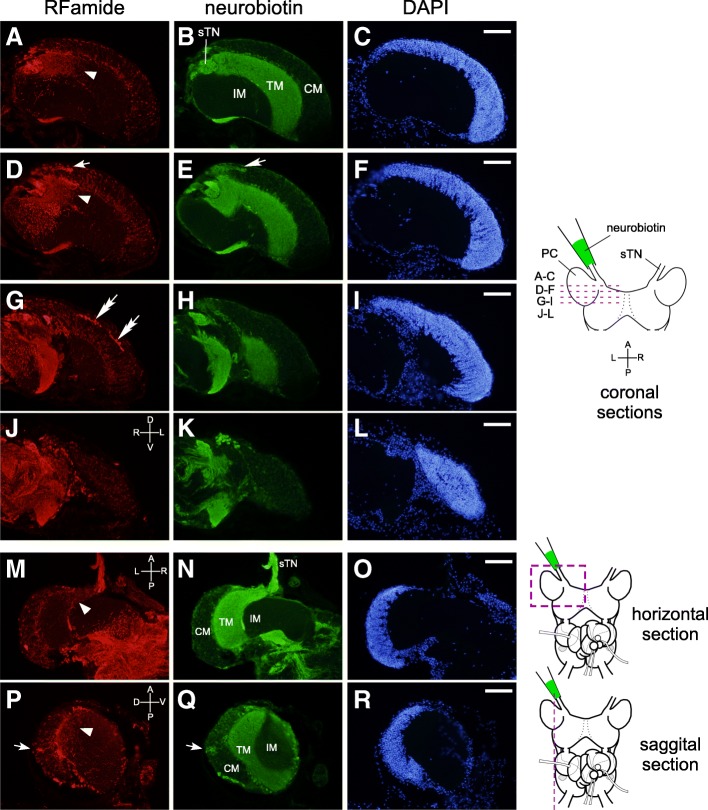
Sections of the PC stained with (**a**, **d**, **g**, **j**, **m**, **p**) anti-RFamide antibody and (**b**, **e**, **h**, **k**, **n**, **q**) streptavidin that binds NB incorporated from the cut end of the superior tentacular nerve. (**c**, **f**, **i**, **l**, **o**, **r**) Fluorescence images of DAPI of respective left panels. White single and double arrows indicate the putative signals of PP and NP-F2, respectively. White arrowheads indicate the RFamide-positive neuropil region in the TM. The schema on the right indicates the cutting plane of the sections. Scale bars: 100 μm (applicable to the photographs in the left). sTN, superior tentacular nerve; CM, cell mass; TM, terminal mass; IM, internal mass, D, dorsal; V, ventral; A, anterior; P, posterior; R, right; L, left

We then examined whether RFamide-containing neurons convey olfactory information from the TG to the PC, or vice versa. NB, a tracer molecule, was incorporated from the cut end of the superior tentacular nerve in an in vitro preparation. Then, the localization of NB and RFamide peptides was visualized histochemically in the CG. Signals of NB were prominent in the terminal mass layer of the PC (Fig. 7b, e, h, n, q), consistent with previous reports [28, 30, 45]. However, RFamide-IR signals were less evident in the terminal mass layer (Fig. 7a, d, g, m, p), except for a few fibers visible in the anterior region of the PC (white arrowheads in Fig. 7a, d, m, p). Similar observations are displayed in Additional file 2: Figure S2A-J.

Further inspection raised the possibility that a few RFamide-IR fibers in the terminal mass layer (white arrowheads in Fig. 7a, d, m, p) derive from cell bodies located in the cell mass layer of the PC (white arrows in Fig. 7d, p). Indeed, these neurons incorporated NB delivered from the cut end of the superior tentacular nerve (white arrows in Fig. 7d, e and p, q), suggesting that these neurons provide efferent projection from the PC to the superior TG. Because the positions of these cell bodies resemble those of PP (see the arrow in the photograph of in situ hybridization of PP in Fig. 3), we speculated that these RFamide-IR fibers were from PP-expressing neurons. We thus generated the antibody that reacts with one of the peptides encoded by PP mRNA, and analyzed the projection patterns of these neurons in the PC.

**Fig. 8:**
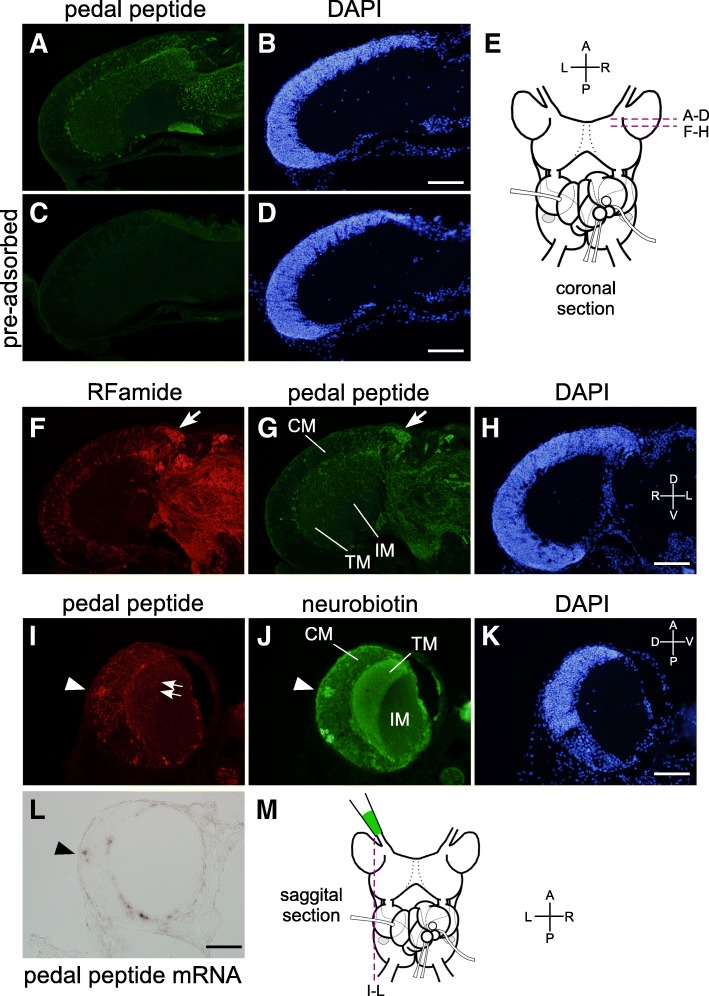
Efferent projection from the PC to the superior TG. **a** Staining of the PC with anti-PP antibody. **c** Pre-adsorption with PP peptide almost diminished the IR signals. **b**, **d** Fluorescence images of DAPI of respective left panels. **e** A schema of the brain showing the cutting plane for (**a**-**d** and **f**-**h**, coronal sections). **f**-**h** Triple staining of RFamide, PP, and nuclei. An arrow indicates the PP-positive cell bodies. **i**-**k** Triple staining of PP, NB, and nuclei in a sagittal section. Arrowheads indicate PP-positive cell bodies that incorporate NB. Arrows indicate the fibers stained with anti-PP antibody. **l** In situ hybridization of PP in a sagittal section. **m** A schema of the brain showing the cutting plane. Scale bars: 100 μm (applicable to the photographs in the left). D, dorsal; V, ventral; A, anterior; P, posterior; R, right; L, left

A pre-adsorption experiment demonstrated the specificity of the antibody (Fig. 8a-d). Dual immunohistochemical staining revealed that this antibody stained a subpopulation of the neurons that were stained with anti-RFamide antibody (compare Fig. 8f and g). Anti-PP antibody also stained some neuronal cell bodies that incorporated NB (arrowheads in Fig. 8i, j). In situ hybridization against PP mRNA in the sagittal brain section further revealed that the position of these cell bodies corresponds to that of NB-incorporating neurons (Fig. 8j, l). Therefore, the PP-expressing neurons located at the dorso-medial position in the PC seem to incorporate NB that was delivered from the cut end of the superior tentacular nerves, suggesting that these PP-expressing neurons provide efferent projection back to the ST.

## Discussion

The generation of the synchronous oscillation of the LFP has been explained by the reciprocal synaptic interaction between the two types of neurons: the predominant nonbursting neurons and a smaller number of bursting neurons [31, 60]. In this framework, the bursting neurons suppress the nonbursting neurons around them with glutamate, and the nonbursting neurons in turn give feedback excitatory cholinergic input to the bursting neurons. Many of the PC neurons have been demonstrated to express vesicular glutamate transporter (a molecular marker of glutamatergic neurons) and vesicular acetylcholine transporter (a molecular marker of cholinergic neurons) [40, 44].

However, a more complex heterogeneity of the PC neurons has recently been proposed in a few reports. For example, many PC neurons express various types of nicotinic acetylcholine receptor variants depending on their location within the PC, and the neurons in the apical region of the PC express high levels of acetylcholine esterase and low levels of vesicular glutamate transporter [40, 44]. Moreover, anti-FMRFa antibody stained a subpopulation of neurons in the PC, and their signal intensities were not the same among the PC neurons [32], suggesting that the expression level or the molecular species of RFamide peptides is also heterogeneous.

In the present study, we revealed the whole picture of the distribution of RFamide-encoding mRNAs in and near the PC by systematic in situ hybridization, and demonstrated an unexpected heterogeneity of the PC neurons with respect to the neuropeptides they use. Ten different RFamide-encoding mRNA species were identified in the PC with various spatial expression patterns (LFRFa1, LFRFa2, CCK/SK1, CCK/SK2, NP-F1, NP-F2, PP, FaRP-B, luqin1, and luqin2, Fig. 3). If all the PC neurons are categorized into either the bursting or nonbursting neurons [60], it is expected that the currently identified RFamide peptides coexist with glutamate or acetylcholine. Future studies should clarify this point by analyses of co-localization of neurotransmitters (e.g. by dual fluorescence immunohistochemistry).

In our analysis, we first determined the positions of the neurons expressing canonical FMRFa mRNA. A cRNA probe was directed to the first exon that is known to be shared by two alternative splice variants [3, 32, 52]. Therefore, the expression patterns of the two variants were not distinguished in the present study. The distribution pattern of FMRFa mRNA was different from the IR detected with FMRFa antibody reported in Kobayashi et al. [32], consistent with the low specificity of anti-FMRFa antibody as has been suggested previously [20]. At the same time, the distribution pattern of FMRFa mRNA in the CG was also considerably different from the IR detected with pan-RFamide antibody (Figs. 3, 7 and 8, Additional file 2: Figure S2). Therefore, FMRFa-expressing neurons comprise a minor subpopulation in the PC, and the effect of exogenously applied FMRFa to LFP oscillation, observed in Kobayashi et al. [32], would reflect FMRFamidergic innervation from outside of the PC, as in the case of serotonin [10].

GSLFRFa is predicted to be encoded in two different transcripts, LFRFa1 and LFRFa2, both of which are abundantly expressed in the PC (Figs. 2b and 3). Therefore, the observed electrophysiological effect of GSLFRFa on the LFP oscillation (Fig. 6) may mimic the effect of this peptide derived from the interior of the PC. Given that FMRFa seems to exert its effect via projections from outside of the PC, the sources of RFamide family peptides acting on the PC reside both inside and outside of the PC.

In the context of previous reports on the neuropeptides that we focused on in the present study, some of their expression patterns in the CG were consistent with expectations. For example, luqin1 and luqin2 were predicted to encode a peptide similar to cardioexcitatory peptide isolated from *Achatina fulica*, which has excitatory action on penis retractor muscle [13]. Of these two genes, luqin1 exhibited prominent expression in the mesocerebrum (Fig. 3), where the neurons controlling mating behavior reside in pulmonates [34]. Satake et al. [53] have actually reported the dominant expression of the putative homologue of this gene in the right CG in *Achatina fulica*, probably reflecting the expression in the mesocerebrum. On the other hand, the expression of NP-F2, LFRFa1and LFRFa2 in the PC was somewhat unexpected because the putative homologues of these genes in *Aplysia* encode neuropeptides that regulate the feeding central pattern generator in the buccal ganglion [26, 57]. Thus far, the PC’s direct involvement in the feeding pattern generation is unknown. These neuropeptides might have quite distinct roles in the olfactory information processing in the PC of pulmonates because of their characteristic expression patterns (Fig. 3).

More than half of the RFamide-encoding mRNA species exhibited dominant expression in the postero-medial (i.e., basal) region of the PC (LFRFa1, CCK/SK1, CCK/SK2, NP-F1, NP-F2, FaRP-B, luqin1, luqin2, Fig. 3). This region of the PC may be involved in some roles distinct from those other PC olfactory interneurons play, because vesicular glutamate transporter and vesicular acetylcholine transporter mRNAs are expressed at a very low level in this region, indicating these neurons are neither bursting nor nonbursting neurons [40, 44]. The expression of CCK/SK1 and CCK/SK2 was most conspicuous there. These two genes encode neuropeptides sharing high similarity to each other (86.6 and 100%, respectively, Fig. 1), and all of them contain the DY motif characteristic of insects’ sulfakinin peptides, which have contractile effects on the muscles of digestive tracts and heart [49]. The physiological role of these peptides is not known in gastropods, and to answer whether these peptides are involved in olfactory information processing awaits future studies.

Most of the RFamide peptides predicted to be encoded in FaRP-A mRNA were also encoded by type-I variant of FMRFa mRNA (Fig. 1). However, the number of positive neurons was far smaller in the CG, and the localization of the cell bodies of FaRP-A-expressing neurons was distinct from that of FMRFa-expressing neurons. It will be intriguing to learn whether these two genes have distinct roles in the brain of the slug.

Although two of the three RFamide peptides predicted to be encoded in FaRP-B (QLHNFVRFa, Fig. 1) were also encoded by FaRP2 of *Deroceras* [1], FaRP-B precursor protein showed only moderate identity (53%) at the amino acid sequence level with FaRP2 of *Deroceras*. The expression was restricted to the dorsal aspect of the CG, and the total number of the positive neurons was only 6 (Fig. 3). As the expression of FaRP-B seemed more abundant in the SEG (Fig. 2b), the peptides encoded by this mRNA may primarily function in motor or endocrine neurons.

We also determined the distribution of RFamide-encoding mRNAs in the ST/IT (Figs. 4 and 5). The relative number of those peptidergic neurons and their locations were similar between the superior and inferior TGs as a whole. However, the relative number of positive neurons in the TGs does not necessarily correlate with that in the PC. For example, the number of neurons expressing PP were moderate in the TGs (although many photoreceptors in the eye seemed to express this gene), whereas those in the PC were numerous. Moreover, there were numerous neurons expressing NP-F1 in the superior/inferior TGs, whereas only a small number of neurons expressed this gene in the PC. One possible explanation for the difference in the composition of RFamidergic neurons may be due to the difference in the sensory modality to which the TGs and the PC are devoted. The TGs are involved in mechanosensation in addition to olfaction, whereas the direct involvement of the PC in mechanosensation is so far not documented [4, 6, 23, 25].

In the terminal mass layer of the PC, RFamide-IR signals could be seen only sparsely in the dorso-anterior region (Figs. 7a and 8i), and most of them seem to be PP-containing processes derived from the PC (Fig. 8). We also observed in our previous study that the tentacle amputation did not diminish the FMRFa-like IR in the terminal mass layer of the PC, supporting the view that these signals reflect those derived from the PC rather than the TGs [41]. Therefore, the RFamidergic projection from the TGs seemed to bypass the PC, and enter directly into the metacerebrum (Fig. 7a, Additional file 2: Figure S2K). These direct pathways from the TG to the metacerebrum convey excitation in advance of the transmission to the terminal mass layer of the PC, and are thought to transmit mechanosensory information [23, 38]. The RFamidergic fibers in the tentacular nerves, therefore, may be primarily involved in the transduction of mechanosensation to the brain. Future studies will be needed to uncover by what neurotransmitters olfactory information is conveyed from the TG to the PC. At the same time, it is unlikely that RFamides in the TG are involved only in the transmission of mechanosensory information to the brain. Rather, they may take part in olfactory information processing within the TG as well. The functional roles of RFamide peptides in the TG represent an intriguing issue to be investigated in the future.

The precursor of PP proved to be a large protein (1487 aa), and was predicted to be cleaved into as many as 42 neuropeptides, two of which are RFamides (Fig. 1). There was not an apparent homologue of PP in the report of *Deroceras* neuropeptides [1]. In Genbank, we found several data of precursor proteins whose amino acid sequence was similar to *Limax* PP, such as pedal peptide precursor protein in *Helix lucorum* (AAB51694) and FMRFa neuropeptide-like precursor in *Charonia tritonis* (AQS80506, Bose et al., [2]) in addition to pedal peptide precursor protein in *Lymnaea* (AAP57098, Hoek et al., [21]). However, the similarity was restricted to the C-terminal half of *Limax* PP. The sole data of amino acid sequence similar to *Limax* PP in the whole protein’s region was found in the transcriptome data of *Aplysia* (XP_005098921, Moroz et al., [47]), although little annotation has been assigned to this protein. The paucity of full-length data on this protein’s homologues in Genbank may be due to the difficulty in the PCR cloning of the cDNAs with highly repetitive sequences [22]. Because neuropeptides are often encoded in multiple copies in a single precursor protein in gastropods [37, 54, 56], there still may be many other genes that have yet to be cloned as full-length transcripts.

We do not exclude the possibility that some cDNAs encoding RFamide peptides may have evaded our computational screening. This is partly because we translated the cDNA sequences derived from our *Limax* transcriptome data only in the sense direction during the computational screening, i.e. only in three frames, which would potentially overlook the data of the antisense cDNA strand, which might be contained to some extent. Moreover, the transcriptome data we initially used (see Materials and Methods) did not contain the data from the PC or the ST/IT. However, we further improved the coverage by also using our personal transcriptome data of the whole brain and the ST (unpublished observation). On the other hand, there also remains the possibility, although unlikely, that the cDNAs encoding RFamides that were not flanked with canonical dibasic amino acids (Arg/Lys-Arg/Lys) but with monobasic amino acids (either of Arg or Lys) were overlooked. In the present study, we successfully identified 11 homologues of 11 *Deroceras* RFamide genes [1], although it is uncertain whether *Limax* FaRP-B is actually the orthologue of *Deroceras* FaRP2. Furthermore, we also found FaRP-A and PP as thus far unidentified RFamide genes. Therefore, our screening would have succeeded in covering a substantial part of the total RFamide transcripts.

It should be noted that FxRIamide (Phe-X-Arg-Ile-NH_2_) genes were excluded from our present study because the N-termini of the encoded peptides were RIamide rather than RFamide, although two FxRIamide genes have been categorized as RFamide genes by Ahn et al. [1].

The data from NB incorporation experiments strongly imply efferent projections from the PP-expressing PC neurons back to the ST (Figs 7 and 8), although, strictly speaking, it is also possible that those PC neurons form electrical synapses with afferent projection nerves from the superior TG somewhere between the TG and the PC. If these PC neurons are indeed providing backward projections to the TG, they may play critical roles in the reciprocal communication between the TG and the PC. Several studies have demonstrated that the engrams of olfactory memory reside not only in the PC but also in the TG that was used during the memory formation, because the functioning of the tentacle that was used during the memory acquisition is necessary for the memory to be retrieved [12, 33]. Therefore, it is possible that those PP-expressing neurons participate in the memory engram encompassing the TG and the PC.

We also observed another cluster of PC neurons, located in the posterior area of the CM layer, that reproducibly incorporate NB (Figs. 7k, q and 8j). A majority of these neurons were not stained with anti-pan-RFamide (Fig. 7j, p) or anti-PP (Fig. 8i) antibodies. Therefore, the PP-expressing neurons would not be the sole PC neurons that send projections back to the TG. A cross-interaction between the TG and the PC might be much more complex than previously thought.

## Conclusions

The roles of RFamide peptides in gastropods have been studied primarily in the context of the actions on effector muscles or on large identifiable neurons. Less attention has been paid to RFamide peptides in the PC of terrestrial gastropods because the PC has been considered to be a neuronal cluster consisting of numerous small interneurons of only a few types. Our present findings have led us to revise this view, and demonstrate that the PC consists of an unexpectedly wide variety of neurons with respect to their component neuropeptides.

## Additional files

Additional file 1: **Figure S1.** Specificities of the RNA probes used in in situ hybridization shown by the absence of signals with sense probes in the adjoining sections. Scale bar: 100 μm (applicable to all photographs). D, dorsal; V, ventral; R, right; L, left. (PDF 63741 kb)

Additional file 2: **Figure S2.** Immunostaining of RFamide in the horizontal sections of the cerebral ganglia. Left hemiganglion is shown. (B, D, F, H, J, L, N) Fluorescence images of DAPI of (A, C, E, G, I, K, M). A magnified merged image of RFamide-IR and DAPI is shown as an inset in (C) and (E). Arrowheads indicate positively stained cell bodies in the metacerebrum and mesocerebrum. Note that not all the positive neurons are marked. White double arrows indicate positively stained nerves in the superior tentacular nerves. Arrowheads indicate the IR signals in the cell bodies, and the putative signals of luqin1, NP-F2 and PP are indicated by large arrowheads marked with “X”, “Y”, “Z”, respectively. (O) Schematic drawings indicating the photographed area (dorsal view, *left*) and the cutting planes (anterior view, *right*). Scale bar: 200 μm (applicable to all photographs). sTN, superior tentacular nerve; PC, procerebrum; R, right; L, left; A, anterior; P, posterior; D, dorsal; V, ventral. (PDF 26260 kb)

Additional file 3: **Figure S3.** Immunostaining of RFamide in the horizontal sections of the left tentacles. *Left*; IR signals of RFamide in the ST. (B, D, F, H) Fluorescence images of DAPI of (A, C, E, G). *Right*; IR signals of RFamide in the IT. (J, L, N, P) Fluorescence images of DAPI of (I, K, M, O). Arrows indicate positively stained cell bodies in the superior or inferior TG. Those near the olfactory epithelium are indicated by arrowheads. Note that not all the positive neurons are marked. White double arrows indicate positively stained nerves in the superior or inferior tentacular nerves. Below is the schema showing the horizontal cutting planes. Scale bar: 200 μm (applicable to all photographs). R, right; L, left; A, anterior; P, posterior; D, dorsal; V, ventral. (PDF 28485 kb)

## Abbreviations

CCD: charge-coupled device
CCK/SK: cholecystokinin/sulfakinin
CG: cerebral ganglia
CM: cell mass
CNS: central nervous system
DAPI: 4′6-diamidino-2-phenylidole
FaRP: FMRFamide-related peptide
FMRFa: Phe-Met-Arg-Phe-NH_2_
FxRIamide: Phe-X-Arg-Ile-NH_2_
IM: internal mass
IR: immunoreactive immunoreactivity
IT: inferior tentacle
LFP: local field potential
LFRFa: Leu-Phe-Arg-Phe- NH_2_
NB: neurobiotin
PBS: phosphate-buffered saline
PC: procerebrum
RFamide: Arg-Phe-NH_2_
RT-PCR: reverse transcription-polymerase chain reaction
SEG: subesophageal ganglia
ST: superior tentacle
TG: tentacular ganglion
TM: terminal mass
