# Supplementary figures and images for: RFamidergic neurons in the olfactory centers of the terrestrial slug Limax
Source: Zoological Lett. 2018 Aug 9;4:22. doi: 10.1186/s40851-018-0108-9 (PMC6085721; doi:10.1186/s40851-018-0108-9)

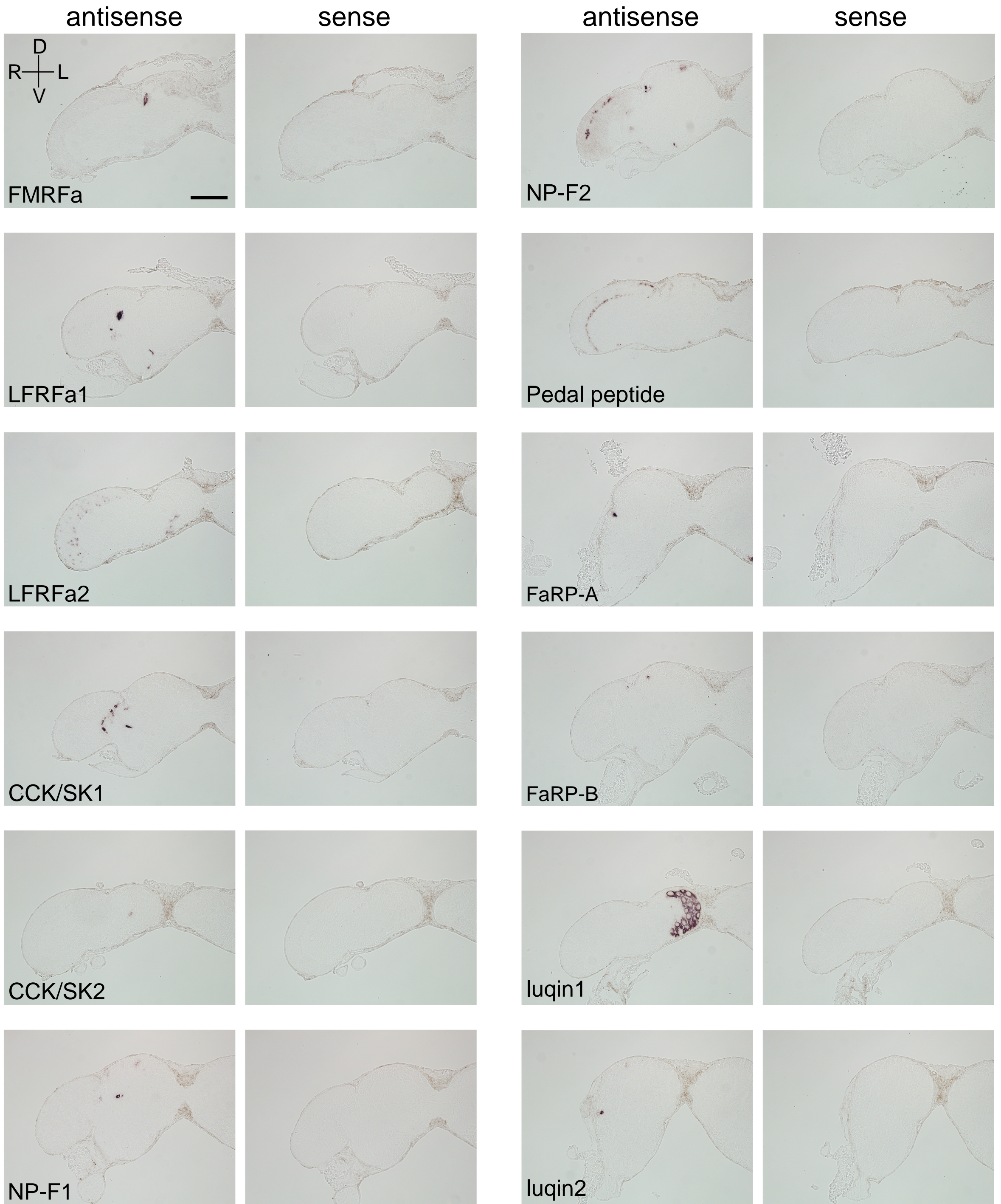

Figure S1

Supplement: Supplementary file 1 — Figure S1. Specificities of the RNA probes used in in situ hybridization shown by the absence of signals with sense probes in the adjoining sections. Scale bar: 100 μm (applicable to all photographs). D, dorsal; V, ventral; R, right; L, left. (PDF 63741 kb) [file 40851_2018_108_MOESM1_ESM.pdf]

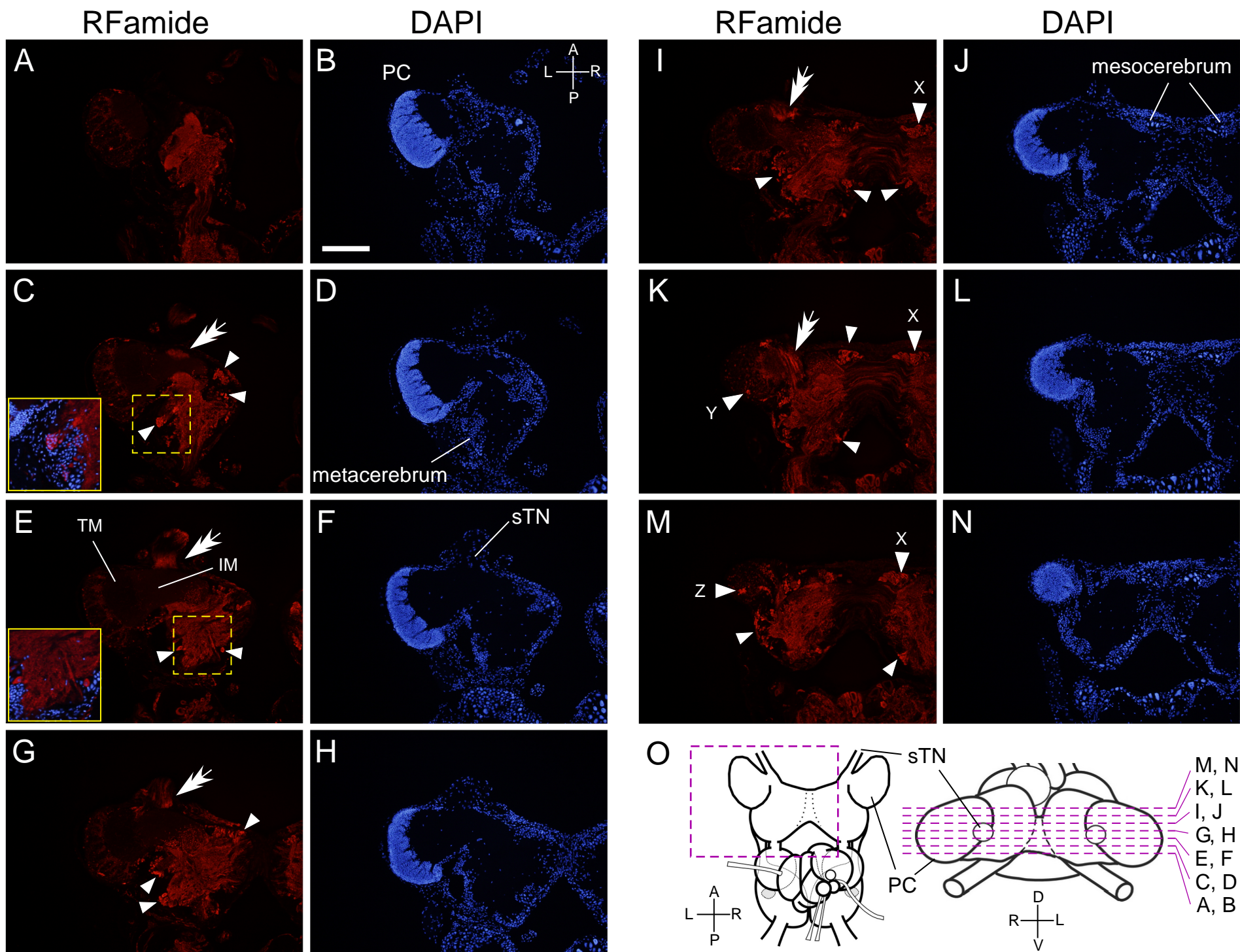

Figure S2

Supplement: Supplementary file 2 — Figure S2. Immunostaining of RFamide in the horizontal sections of the cerebral ganglia. Left hemiganglion is shown. (B, D, F, H, J, L, N) Fluorescence images of DAPI of (A, C, E, G, I, K, M). A magnified merged image of RFamide-IR and DAPI is shown as an inset in (C) and (E). Arrowheads indicate positively stained cell bodies in the metacerebrum and mesocerebrum. Note that not all the positive neurons are marked. White double arrows indicate positively stained nerves in the superior tentacular nerves. Arrowheads indicate the IR signals in the cell bodies, and the putative signals of luqin1, NP-F2 and PP are indicated by large arrowheads marked with “X”, “Y”, “Z”, respectively. (O) Schematic drawings indicating the photographed area (dorsal view, left) and the cutting planes (anterior view, right). Scale bar: 200 μm (applicable to all photographs). sTN, superior tentacular nerve; PC, procerebrum; R, right; L, left; A, anterior; P, posterior; D, dorsal; V, ventral. (PDF 26260 kb) [file 40851_2018_108_MOESM2_ESM.pdf]

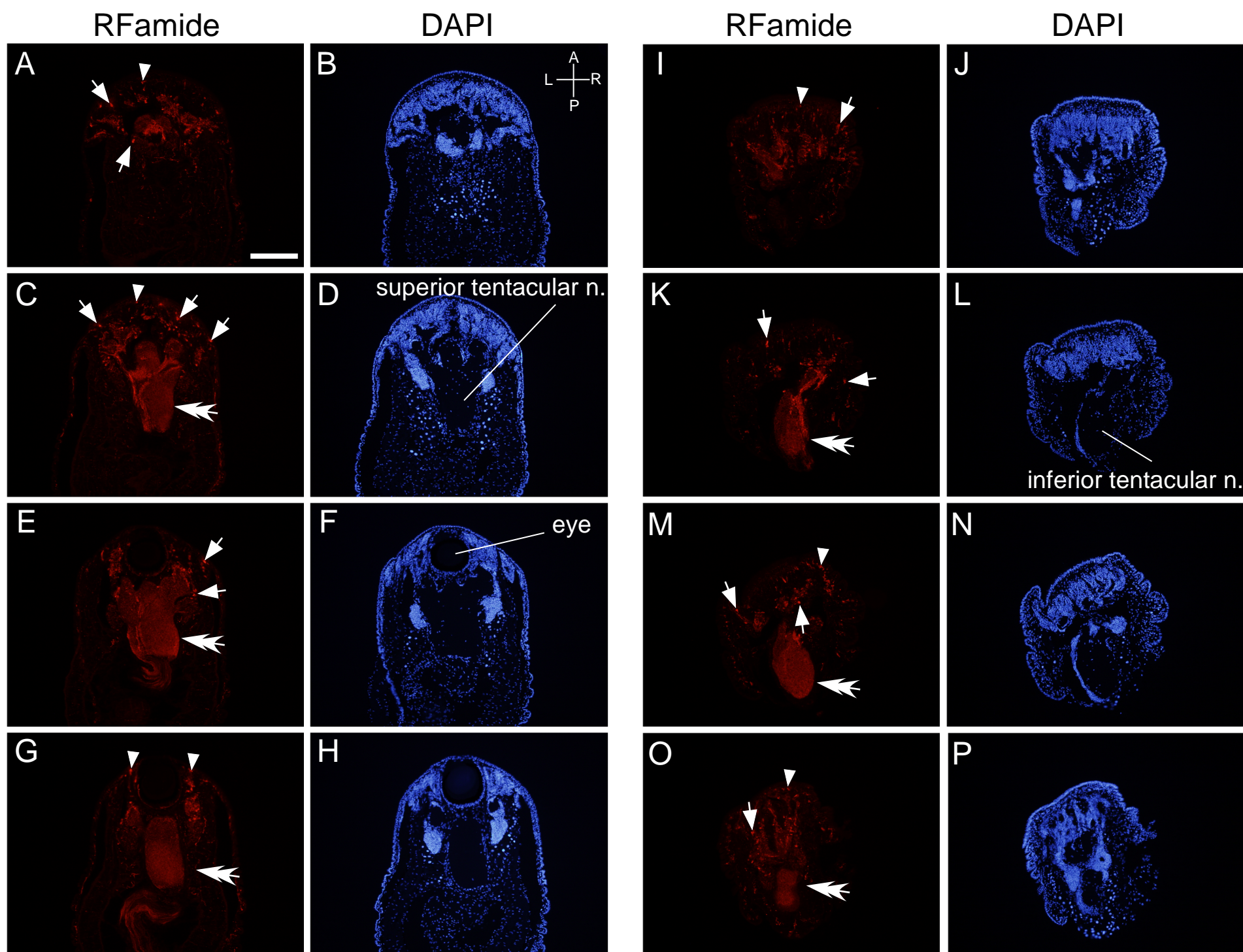

superior tentacle

inferior tentacle

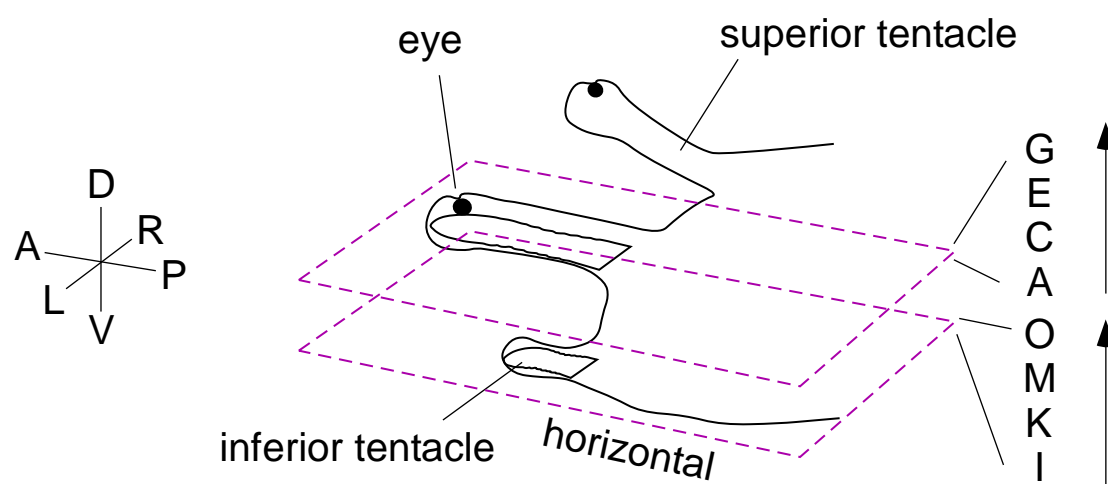

Figure S3

Supplement: Supplementary file 3 — Figure S3. Immunostaining of RFamide in the horizontal sections of the left tentacles. Left; IR signals of RFamide in the ST. (B, D, F, H) Fluorescence images of DAPI of (A, C, E, G). Right; IR signals of RFamide in the IT. (J, L, N, P) Fluorescence images of DAPI of (I, K, M, O). Arrows indicate positively stained cell bodies in the superior or inferior TG. Those near the olfactory epithelium are indicated by arrowheads. Note that not all the positive neurons are marked. White double arrows indicate positively stained nerves in the superior or inferior tentacular nerves. Below is the schema showing the horizontal cutting planes. Scale bar: 200 μm (applicable to all photographs). R, right; L, left; A, anterior; P, posterior; D, dorsal; V, ventral. (PDF 28485 kb) [file 40851_2018_108_MOESM3_ESM.pdf]
